# Range-wide and temporal genomic analyses reveal the consequences of near-extinction in Swedish moose

**DOI:** 10.1038/s42003-023-05385-x

**Published:** 2023-10-17

**Authors:** Nicolas Dussex, Sara Kurland, Remi-André Olsen, Göran Spong, Göran Ericsson, Robert Ekblom, Nils Ryman, Love Dalén, Linda Laikre

**Affiliations:** 1 Centre for Palaeogenetics, Svante Arrhenius väg 20C, SE-106 91 Stockholm, Sweden; 2 Department of Zoology, Division of Population Genetics, Stockholm University, SE-106 91 Stockholm, Sweden; 3 Department of Bioinformatics and Genetics, Swedish Museum of Natural History, SE-104 05 Stockholm, Sweden; 4 Norwegian University of Science and Technology, University Museum, Trondheim, NO-7491 Norway; 5 Science for Life Laboratory, Department of Biochemistry and Biophysics, Stockholm University, SE-171 21 Solna, Sweden; 6 Department of Wildlife, Fish, and Environmental Studies, Swedish University of Agricultural Sciences, SE-901 83 Umeå, Sweden; 7 Wildlife Analysis Unit, Swedish Environmental Protection Agency, SE-106 48 Stockholm, Sweden

**Keywords:** Genetic variation, Population genetics

## Abstract

Ungulate species have experienced severe declines over the past centuries through overharvesting and habitat loss. Even if many game species have recovered thanks to strict hunting regulation, the genome-wide impacts of overharvesting are still unclear. Here, we examine the temporal and geographical differences in genome-wide diversity in moose (*Alces alces*) over its whole range in Sweden by sequencing 87 modern and historical genomes. We found limited impact of the 1900s near-extinction event but local variation in inbreeding and load in modern populations, as well as suggestion of a risk of future reduction in genetic diversity and gene flow. Furthermore, we found candidate genes for local adaptation, and rapid temporal allele frequency shifts involving coding genes since the 1980s, possibly due to selective harvesting. Our results highlight that genomic changes potentially impacting fitness can occur over short time scales and underline the need to track both deleterious and selectively advantageous genomic variation.

## Abstract (web summary)

Collection and analysis of 200 years of Swedish moose genomes reveals regional differences in genetic load, candidate regions for positive selection and an overall limited impact on human-driven bottlenecks on today’s moose population.

## Introduction

Wild species are under increasing pressure from anthropogenic activities with up to 16–33% of all vertebrate species being threatened with extinction^1,2^. Over the past few centuries, many ungulate species have experienced severe population declines through habitat modification and overharvesting and some of them were nearly extirpated from their native range^3^. Management programmes combining habitat restoration, sustainable harvesting and reintroductions aided by the development of wildlife management institutions have allowed several game species to recover^4^, even in regions with high human-wildlife interactions such as Europe^5,6^. Most management plans recognise the role of ungulates in maintaining ecosystem functions as well as their impact on their ecosystem^7,8^. Consequently, ungulate management plans include consideration of population dynamics, migration, multi-ungulate competition, interactions with carnivores and humans, genetics, cascading effects on vegetation, and disease management^6,7^. In practice, these plans often incorporate seasonal hunting limits, quotas and selective harvesting (e.g., trophy hunting)^6^.

However, while the use of genetic tools in conservation programmes has increased rapidly over the past decades^9^, their integration in ungulate management^7^ and in conservation as a whole is still considered weak^10,11^. This is rather surprising because it is well recognised that genetic processes play a crucial role for the long-term survival of species^12–14^. Furthermore, declining populations are exposed to a number of genetic threats, referred to as genomic erosion^15,16^. These threats include loss of adaptive potential limiting the ability to adapt to long-term changes in the environment^13^, but also increase in genetic load (i.e., frequency of harmful mutations^16–18^) through drift and the reduced effect of purifying selection, maladaptation (i.e., mismatch between adaptations and environment), and genetic introgression after hybridisation^16,19^. Estimation of parameters associated with genomic erosion is thus increasingly considered as a major component of species conservation programmes^15,20^.

Conservation and management of wild game species have greatly benefited from decreasing costs in sequencing^21^ and from a growing number of genomic resources being generated for rare and threatened species^22^. These resources allow to reconstruct species’ demographic fluctuations and to estimate temporal and geographical changes in selection regimes, genome-wide diversity, and the amount of genetic load^15,20^. Several initiatives have advocated for a better use of genetics in conservation^23^. For instance, the new Global Biodiversity Framework of the Convention on Biological Diversity (CBD; www.cbd.int) includes goals to maintain genetic diversity of populations of all species to secure their adaptive potential. To secure this goal, the use of DNA-based techniques and the integration of quantitative goals to safeguard the retention of genetic variation in species management programmes are needed^23–26^. The new CBD framework also includes a monitoring framework with a Headline indicator for genetic diversity focusing on effective population size (N_e_) and Essential Biodiversity Variables (EBVs) that include genome-wide diversity, inbreeding and gene flow, which are becoming increasingly important to monitor trends of genetic diversity ^26,27^. Estimating those indicators and other genomic parameters in intensively hunted game species would thus allow to assess the genomic impacts associated with natural disturbances or intensive hunting and greatly improve their long-term monitoring and management^22,27^.

A growing number of empirical studies have investigated the genomic consequences of near-extinction in the wild and have illustrated the complex dynamics of genetic load in small populations (e.g.,^28–30^). For instance, the magnitude and speed of a decline and recovery, as well as life-history traits, will have a strong impact on genetic load and of its components^31,32^. Theoretical predictions and empirical data indicate that purifying selection is most efficient at purging highly deleterious mutations^31^ while mildly deleterious mutations, which can have a non-negligible impact on fitness^16,33^, tend to accumulate. To date, only a few studies have examined the post-bottleneck genomic variation in game species. For instance, the Alpine ibex (Capra ibex), which had declined to ~100 individuals by the early 1900s and now numbers more than 50,000 individuals spread over most of its former range^34,35^, has lost ~80% of its pre-bottleneck mitochondrial genetic variation^36^. Moreover, a comparison of whole nuclear genomes of Alpine ibex and other caprinae indicates that after this historical bottleneck, the strong founder effects associated with reintroductions facilitated the purging of its highly deleterious genetic variation whereas mildly deleterious mutations accumulated^37^.

Reduction in adaptive potential or changes in allele frequency for genes relevant to individual fitness will also impact the likelihood of long-term survival of species^9,38^. In a rapidly warming world, identifying adaptive variation and tracking its changes through time is essential for species conservation^26^. For instance, evidence for local adaptation to distinct environments requires the designation of appropriate management units to maximise the long-term maintenance of evolutionary potential of species^26,27^. This type of information is crucial in the context of translocations, genetic rescue, or assisted gene flow^39^. Moreover, tracking changes in adaptive variation could inform us on the ability of species to adapt to changing environmental conditions^40,41^ or on the unintended effects of management or hunting practices^21^. Genome-scans of selection are thus often used to identify outlier genomic regions that are putatively under positive selection due to local adaptation^42^.

Moose (*Alces alces*) populations were severely reduced in Fennoscandia from the 15th century onwards^43^. Intensification of hunting during the 18th and 19th centuries led to near-extinction of moose in Fennoscandia^44^. Moose were rare outside South-central Scandinavia at the time and harvesting data from the 1890s onwards suggest that the population numbered in the low thousands^45,46^. Since the 1930s, increased commercial forestry has benefitted the moose^43^ and hunting legislation allowed the species to recover and its harvest is now intensively regulated^47^. The Swedish population currently numbers ~350,000 individuals and ~1/3 of its population is harvested annually^48^. In spite of this near-extinction event, the Swedish moose population has relatively low inbreeding levels relative to North American populations, which could be explained by rapid recolonisation of the range from isolated and less impacted populations in Norway or Finland after the near-extinction event^49^. However, Scandinavian moose also has one of the lowest genetic diversity in Europe and shows a high degree of isolation and genetic differentiation relative to other Fennoscandian populations^50–52^. Furthermore, such a severe decline to near -extinction suggests that the pre-decline diversity could have been much higher, and that the moose population may be exposed to genome erosion. Furthermore, there is evidence for geographical variation in body size and antler morphology at the extremes of moose range possibly associated with local adaptation to varying habitat types^53,54^. The Swedish moose population is thus an ideal model species to develop and test genomic tools for game management and monitoring^55^.

Here, we use temporally-spaced genomes collected over the past ~200 years across the whole range of moose in Sweden and examine the geographical and temporal differences in genome-wide diversity resulting from the recent human-driven near-extinction. We also estimate recently adopted genomic indicators relevant to the long-term monitoring of genetic diversity in wild populations^24^ and examine geographical and temporal patterns of genome differentiation. Our data shows limited impact of the recent bottleneck on the modern moose population. However, we found evidence for regional differences in genetic load while genomic indicators raise some concerns for future reduction in gene flow and genetic diversity. Finally, we found candidate regions for positive selection due to local adaptation when comparing the extremes of the range and as well as temporal genetic differentiation in regions containing genes potentially relevant to individual fitness.

## Results

### De-novo assembly

We improved the previous assembly from Dussex et al.^49^. (GenBank accession: GCA_015832495.1) from a sample of the same female individual and by adding long-read Hi-C scaffolding. The new moose assembly has a scaffold N50 of 76.6 Mb with 99% of the assembly composed of 34 scaffolds, suggesting that our assembly is at or close to chromosome-level. A total of 8660 out of 8771 single-copy mammalian BUSCOs were found within these 34 scaffolds. A synteny analysis against red deer (*Cervus elaphus*) allowed us to identify 32 autosomes as well as the X and Y chromosomes (HiC_scaffold_21, HiC_scaffold_24; Supplementary Data 1). In contrast, the red deer assembly had 33 autosomes and two sex chromosomes, suggesting few chromosomal rearrangements in moose.

### Population structure and past demography

**Fig. 1:**
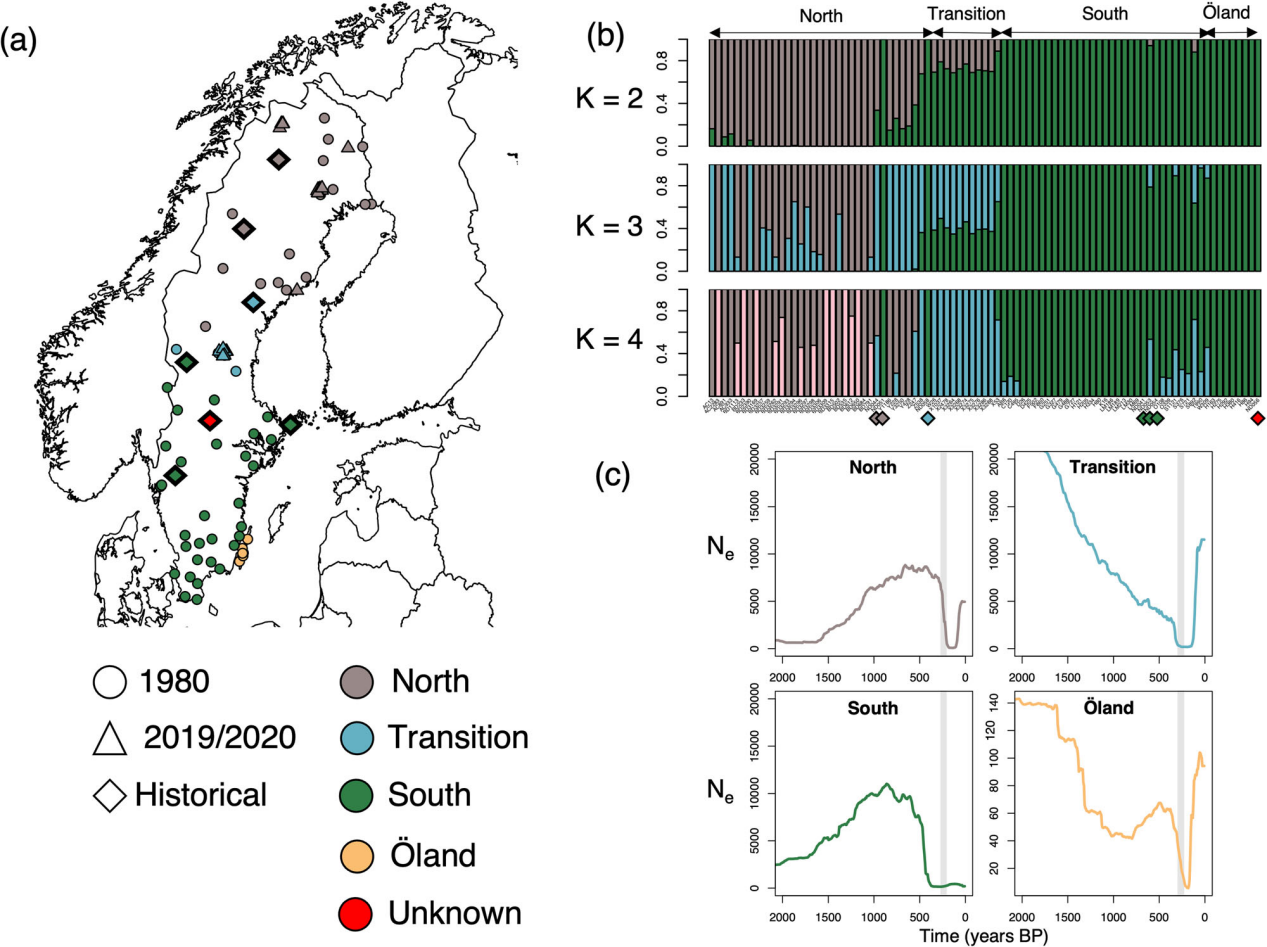
Sampling, population structure and past demography of Swedish moose. **a** Sampling locations for 87 genomes including the seven historical genomes (1839-1905; Supplementary Data 2). Populations include the three genetic clusters identified by Wennerström et al.^48^ and Öland. **b** Admixture plot for moose genomes for K = 2-4. **c** Demographic reconstruction of effective population size (N_e_) using the Linkage Disequilibrium approach implemented in GONE. N_e_ curves depict the geometric mean over 40 independent estimates. The X-axis represents time before present in years, assuming a generation time of seven years^49^ and the Y-axis depicts N_e_. Vertical grey bars depict approximate timing of hunting intensification ~200–300 years BP.

We sequenced 87 genomes at ~17X depth of coverage (historical: 9X; 1980: 16X; 2019/2020: 22X) from across the whole range of moose in Sweden (Supplementary Data 2). We found evidence for isolation by distance in the form of a cline as shown in Wennerström et al.^48^. We obtained the highest support for K = 3 (Fig. 1b) distinguishing a North, and South cluster as well as an admixed Transition cluster. However, the Transition cluster became fully apparent for K = 4. Genomes from the island of Öland grouped with the South cluster. Out of seven historical specimens (i.e., 1839–1905), six grouped with a cluster corresponding to their sampling location while one, with unknown sampling location (ND056), grouped with the South cluster (Supplementary Data 2).

Past demographic reconstruction using SMC++ indicated a gradual population decline starting ~100,000 years Before Present (BP) from a N_e_ of ~10,000 to ~100 by 500 years BP (Supplementary Fig. 1). However, there was no indication of a bottleneck coinciding with the near-extinction in the 1700s. In contrast, GONE identified in all three clusters a decline starting ~400 years BP with N_e_ being the lowest between 300–140 years BP (Fig. 1c; Supplementary Data 3-4). The island population of Öland showed the lowest N_e_ (i.e., 40–6) during this period.

### Heterozygosity, inbreeding and nucleotide diversity

**Fig. 2:**
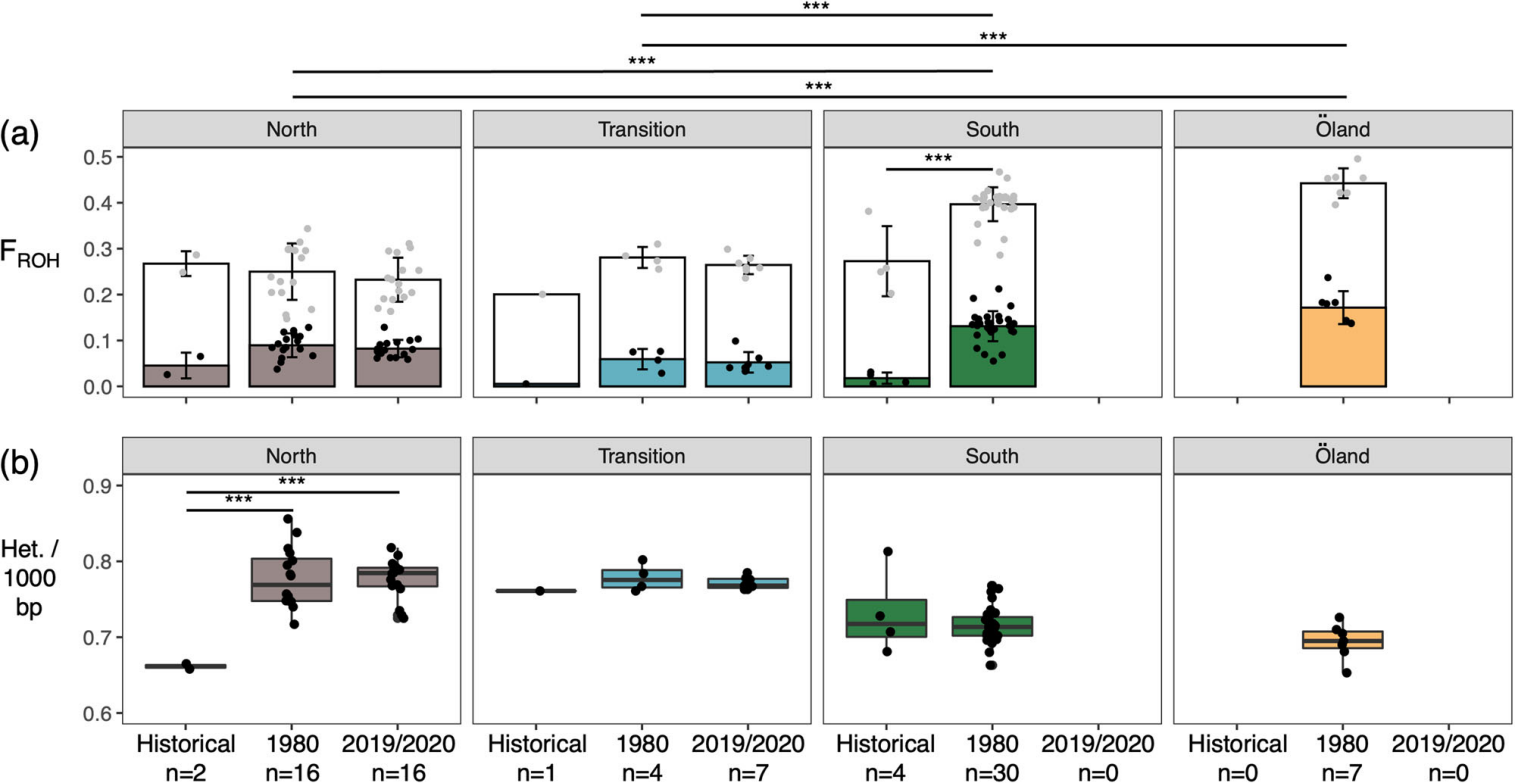
Geographical and temporal comparisons for inbreeding and heterozygosity for 87 Swedish moose genomes. **a** Inbreeding coefficients (F_ROH_). Complete bars show the proportion of genomes in ROH $\ge$ 100 kb (i.e., background relatedness) and lower portions of bars show proportions in ROH $\ge$2 Mb (i.e., recent inbreeding events). **b** Heterozygosity (N. het. sites/1000 bp). Bars extending from the mean values represent the standard deviation. Only significant differences are shown (Tukey’s HSD test; ^***^p < 0.001). Regional significant differences apply to modern populations only.

For modern populations (i.e., samples collected in 1980 and 2019/2020), the data supported a significant increase in inbreeding (F_ROH_) and reduction in heterozygosity (N het. sites/1000 bp) in a southward direction (ANOVA, *n* = 4, *P* = 1.09 × 10^−10^; Fig. 2; Supplementary Data 2), with the island population of Öland showing the highest inbreeding and lowest heterozygosity (Fig. 2a, b; Supplementary Data 2). There was evidence for significant temporal changes in inbreeding within the South cluster including for ROH > 2 Mb between 1800s and 1980s genomes (Tukey’s HSD test, *n* = 2, *P* = 4.3 × 10^−06^; Fig. 2a) and in heterozygosity within the North cluster (Tukey’s HSD test, *n* = 3, *P* = 1.5 × 10^−04^ – 1.8 × 10^−04^; Fig. 2b).

The majority of ROH were <2 Mb long (Fig. 2). Mean ROH length was similar among modern populations (mean length Mb: North = 3.5; Transition = 3.4; South = 3.6; Öland = 3.9; Supplementary Fig. 2), whereas maximal ROH value increased from the north to the south of the range (max. ROH Mb: North= 15.4; Transition = 14.8; South = 30.6; Öland = 23.9; Supplementary Fig. 2). Based on the distribution of ROH length, the majority of inbreeding events in modern genomes (i.e., < 2 Mb) date back to >168 years BP. In contrast, large ROH $\ge$23 Mb in the South cluster and on Öland indicate that inbreeding among individuals with shared ancestry could have occurred as recently as 11 years BP. For historical genomes, most inbreeding events also date back to >168 years before sampling. Thus for samples collected in 1839-1905, the inbreeding events occurred ca. 1670–1740 CE. Moreover, a small number of ROH $\ge$5–10 Mb correspond to inbreeding events occurring between 67 and 33 years before sampling (e.g., for ND044 collected in 1843, the inbreeding event occurred ca. 1780–1800 CE ; Supplementary Fig. 2).

Nucleotide diversity (π) was significantly different among modern populations, with the highest diversity found in the North and Transition clusters (*t* test; *n* = 4, *P* = 2.2 × 10^−16^ < 0.001; Supplementary Data 3). We also found significant temporal changes in π within all clusters (*t* test, *n* = 2-3, *P* = 2.2 × 10^−16^ – 4.37 × 10^−12^; Supplementary Data 3). Within the Northern cluster, π increased between the 1800s and 2019/2020 whereas π decreased within the Transition cluster between 1800s-2019/2020 and in the South cluster between 1800s–1980.

### Genetic load

**Fig. 3:**
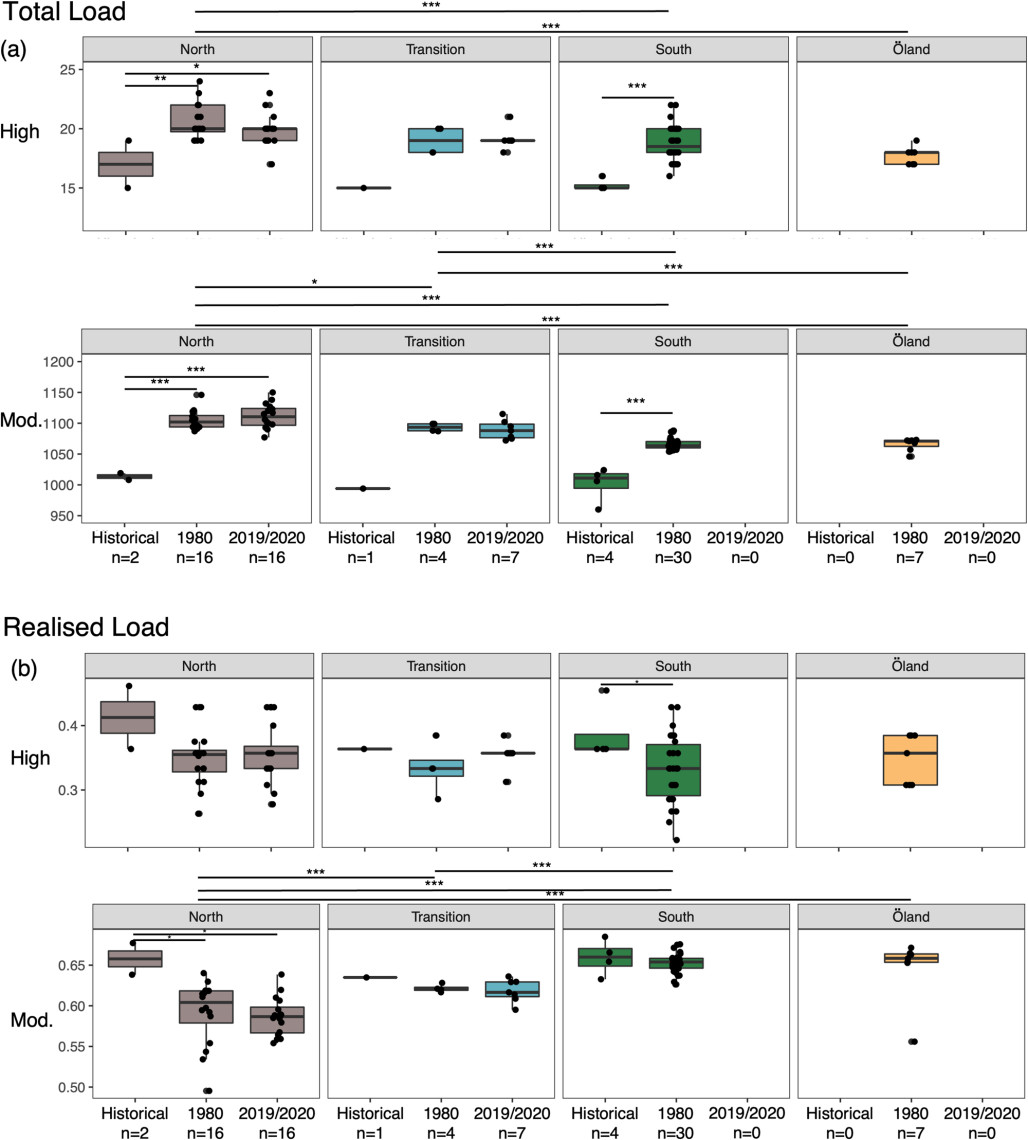
Geographical and temporal comparisons of genetic load in coding regions for 87 Swedish moose genomes. **a** Total counts of High and Moderate impact deleterious variants per individual. **b** Realised load per individualw for High and Moderate impact variants. Bars extending from the mean values represent the standard deviation. Only significant differences are shown (Tukey’s HSD test; ^*^p < 0.05, ^**^p < 0.01, ^***^p < 0.001). Regional significant differences apply to modern populations only.

We quantified genetic load by identifying High and Moderate impacts variants in coding regions. There were significant geographical differences in the total counts of both High (ANOVA, *n* = 4, *P* = 1.28 × 10^−5^) and Moderate (ANOVA, *n* = 4, *P* < 2 × 10^−16^) impact variants among modern populations, with a general reduction in total load towards the south the range (Fig. 3; Supplementary Figs. 3, 4; Supplementary Data 2). While there was no change in genetic load over the past 40 years, we found significant increase in the North and South clusters for the total number of both High (Tukey’s HSD test, *n* = 2-3, *P* < 9.23 × 10^−5^ – 7.7 × 10^−3^; Fig. 3a) and Moderate (Tukey’s HSD test, *n* = 2-3, *P* < 1 × 10^−7^ – 2 × 10^−7^; Fig. 3a) impact variants since the 1800s.

When considering the frequency of variants in each population using R_xy_, there was also evidence for a reduction in load towards the South of the range (Supplementary Fig. 4). Öland showed a reduction in the frequency of High impact but an excess in frequency of Moderate impact variants relative to the South cluster (Supplementary Fig. 4). Furthermore, modern genomes showed an excess in LoF and Moderate impact variation relative to historical ones, indicating a temporal increase in the frequency of deleterious alleles (Supplementary Fig. 4). There were 41 more High impact and 1,339 more Moderate impact variants in modern genomes compared to historical ones, corresponding to 1.7-fold and 1.8-fold increases in those variant categories, respectively (Supplementary Data 5).

We only found significant differences in realised load across the modern range of moose for Moderate impacts variants, with a highest load in the South cluster and on Öland (ANOVA, *n* = 4, *P* = 1.5 × 10^−14^; Fig. 3b). Moreover, realised load estimates indicated significant temporal reductions only in the South cluster for High impact variants (Tukey’s HSD test, *n* = 4, *P* = 0.046; Fig. 3a) and in the North cluster for Moderate impact variants (Tukey’s HSD test, *n* = 4, *P* = 1.8 × 10^−2^ – 2.8 × 10^−2^; Fig. 3b).

Among the genes including High and Moderate impact variants in modern genomes, we identified genes associated with male fertility, hair and skin morphology, bone and eye development, embryogenesis (Supplementary Data 5, 6).

### Genomic indicators

**Fig. 4:**
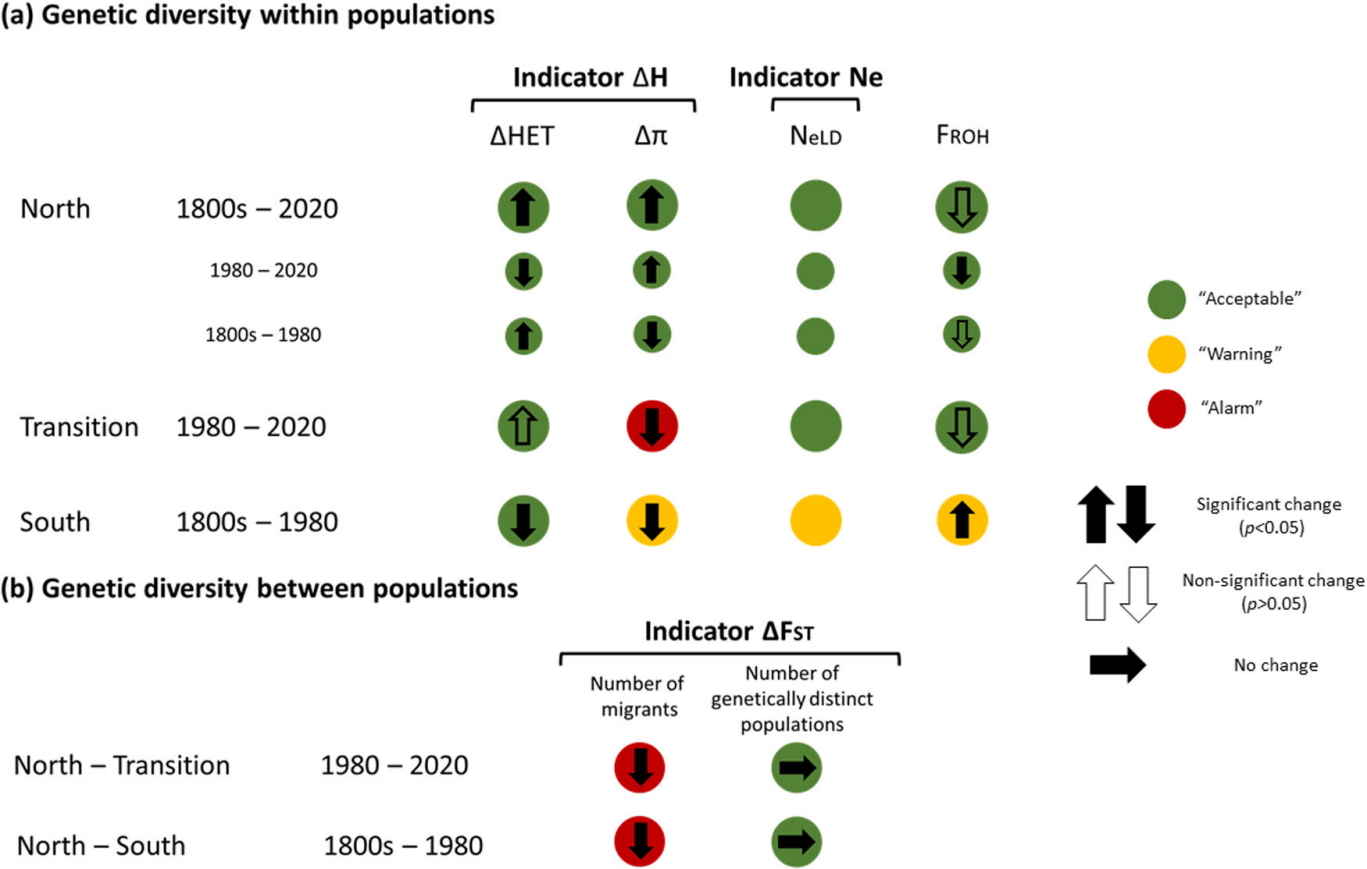
Genomic indicator classifications for 87 Swedish moose genomes. **a** Within population diversity. **b** Between population diversity. Indicators were estimated using the methodology and thresholds from Andersson et al.^24^. Indicators were applied to three genetically distinct Swedish moose clusters. Different temporal comparisons are possible for each population. Three temporal comparisons of genetic diversity within populations are made for the North cluster. ΔF_ST_ was computed for the North cluster compared to the Transition and South cluster.

To assess the status of moose according to CBD recommendations which aim at maximising the retention of genetic variation, we estimated three genetic indicators following Andersson et al.^24^. Diversity within the northern cluster was classified as *Acceptable* for both the ΔH and N_e_ indicators (Fig. 4; Supplementary Data 7). In the Transition cluster, indicator ΔH showed increased heterozygosity and decreased F_ROH_ deemed *Acceptable*, yet an *Alarm* reduction in nucleotide diversity (π). The N_e_ indicator was classified as *Acceptable*. The South cluster showed an *Acceptable* reduction in heterozygosity whereas changes in π and F_ROH_ and N_e_ indicators were classified as *Warning* (Fig. 4; Supplementary Data 7).

A statistically significant temporal increase in F_ST_ was found between the North and the Transition cluster and between the North and South cluster (Wilcoxon signed-ranked test, *n* = 2, *P* = 2 × 10^−16^; Fig. 4, Supplementary Data 8). When translating changes in F_ST_ to gene flow between clusters, both values exceeded threshold values and were classified as *Alarm*. This indicator was classified as *Acceptable* with regard to the maintenance of distinct populations over time, since no populations went extinct during the sampling period.

### Genome scans of selection and gene ontology

**Fig. 5:**
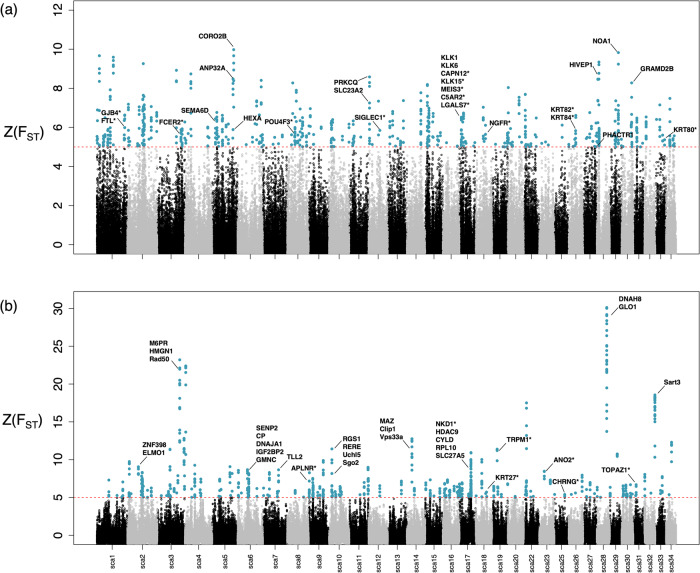
Genome scans of selection in the Swedish moose population. **a** Genome-wide differentiation between the North (*n* = 30) and South (*n* = 30) clusters. **b** Genome-wide differentiation between the 1980 (*n* = 17) and 2020/2019 (*n* = 17) periods for the North cluster. Red dashed lines indicate Z-transformed F_ST_-outlier values outside 5 standard deviations from the mean. Asterisks represent genes expressed in antlers previously identified in Wang et al.^56^.

Genome scans of selection based on Z(F_ST_) values identified 206 candidate genes within outlier windows when comparing the North and South clusters (Fig. 5a; Supplementary Data 9, 10). We identified genes associated with skin pigmentation (e.g., KLHL5, HPS5, RPS21, TEP1; Supplementary Data 9, 10) including three keratin genes (i.e., KRT80, KRT82, KRT84) as well as 14 genes expressed in skin and horns of sheep and goat (e.g., KRT80, KLK15, SIGLEC1, CAPN12) including one gene (NGFR) highly expressed in roe and sika deer antlers^56^. We also found two additional genes from the KLK gene family (KLK1, KLK6) as well as other genes (HIVEP1, PHACTR1, SEMA6D) known to be associated with the cardiovascular system. Finally, we identified outlier genes associated with retinal development (SEMA6D) as well as nervous system (HEXA; Supplementary Data 9, 10).

When testing for association between outlier windows and climate using latitude as a proxy for temperature, 71 out of the 206 candidate genes had variants associated with latitude and included skin pigmentation and antler development genes (e.g., HPS5, FCER2, KLK15, CAPN12, KRT84; Supplementary Data 9, 10).

Temporal comparison between the 1980 and 2019/2020 periods for the North cluster identified 212 candidate genes within outlier windows (Fig. 5b; Supplementary Data 9, 10). Among those, seven genes were associated with antler development (Supplementary Data 9, 10). Among the top outliers windows, we found genes associated with male fertility (e.g., ELMO1, Clip1, Sgo2, GMNC, Dnah8), insulin or fat metabolism (e.g., M6PR, SLC27A5, IGF2BP2), cardiovascular function (e.g., ZNF398, HDAC9, Vps33a, RERE, SENP2) and body weight (e.g., TLL2) (Supplementary Data 9, 10).

## Discussion

We analysed 87 modern and historical genomes over the whole range of moose in Sweden to examine the genome-wide impacts of near-extinction in the 18^th^ century. We found some regional increase in inbreeding and in genetic load since the 1800s, although more samples would be needed to confirm this trend. Importantly, we found a slight but significant southward increase in inbreeding and a reduction in total genetic load but little change in these estimates over the past 40 years. In spite of the weak change in inbreeding and load, we estimated a significant annual reduction in nucleotide diversity over the same period, as well as indications of reduced gene flow highlighting the need for continuous monitoring. Also, effective population size estimate for the modern (1980s) South cluster is below the CBD threshold value of 500, which is cause for concern and calls for an assessment of the present N_e_ of this region. Finally, genome scans of selection identified regions putatively under positive selection due to local adaptation and temporal changes involving genes associated with antler development and other biological functions potentially impacting fitness. These results suggest that the moose population in Sweden is not at immediate risk of exposure to genomic erosion. However, future reduction in gene flow and increases in inbreeding could result in the expression of this genetic load^16^ and harvesting practices could impact functional variation and population fitness^21^.

The population structure described here is consistent with a previous study based on microsatellite data which identified a North-South genetic cline^48^. While this cline could have resulted from colonisation from both the North and South, sharing of rare European mitochondrial haplotypes distinct from the Finnish lineage in the North and South of its range^50,57^ supports a colonisation from the South after the Last Glacial Maximum. Moreover, our data indicates that the island population of Öland belongs to the South cluster and suggests ongoing gene flow. However, even though moose can swim the short distance (~10 km) between the mainland and Öland, non-invasive DNA data suggests that gene flow is probably limited^58^.

Demographic reconstructions supports gradual decline starting ca. 100,000 BP consistent with a previous demographic reconstruction using the PSMC^49^. This decline, which is congruent with a lack of moose fossil remains from Europe during the Last Glacial Maximum ~25,000 years BP^59^, could indicate shifts in the preferred moose habitat characterised by boreal forests and taiga during the cold periods^49^. However, the SMC++ did not detect any reduction in N_e_ associated with hunting intensification of the 18th and 19th centuries. In contrast, the linkage disequilibrium approach in GONE, which outperforms other methods for recent time frames^60^, showed strong support for a demographic decline at that time, as previously shown by Wennerström et al.^48^. We note that the signature of decline on Öland could also correspond to a founder effect.

The overall decline observed here is consistent with a reduction in Major Histocompatibility Complex (MHC) diversity in modern moose populations^61,62^ but is in stark contrast with previous studies on Norwegian and Finnish moose that did not find genetic evidence for reduction in N_e_^63,64^ associated with intensive hunting. This is most likely due to local variation in hunting intensity or alternatively to the lower resolution of markers used in these studies (i.e., microsatellite loci). Interestingly, Öland as well as the Transition and the North clusters show a recent (i.e., ~150 years BP) increase in N_e_, coinciding with the reduction in hunting pressure and the implementation of new hunting regulation in the 1930s^47^.

Estimates of inbreeding were relatively low and ROH distributions were in majority of a length <10 Mb, indicating only a moderate impact of the recent decline. However, the southward increase in F_ROH_ in modern moose and maximum ROH length in the South cluster is consistent with a more intense bottleneck in that part of the range resulting from either a founder effect after recolonisation of this area at the beginning of the 20th century or a strong hunting pressure over the past decades^65^. In contrast, while the Öland population is small (i.e., 95% CI: 115–156) and the most inbred population in Sweden, the management strategy and reduced number of individuals harvested ranging from 0 to 43 individuals per year, suggests that the main causes of the high inbreeding are the founder effect and reduced gene flow^58^.

We found little evidence for temporal changes in F_ROH_ or heterozygosity since the 1800s, except in the South and North clusters. We caution that more genomes would be required to confirm the trend observed between the 1800s and 1980s. Yet, inbreeding estimates based on single genomes can be highly informative in inferring the timing and magnitude of past bottlenecks (e.g., Woolly rhinoceros, *Coelodonta antiquitatis*^66^). Since recombination breaks ROH into smaller fragments, large fragments reflect recent inbreeding events, whereas smaller ones reflect older inbreeding events^67^. Here, the distribution of ROH indicates that most inbreeding events occurred >168 years prior sampling of historical and modern genomes, and thus date back to ca. 1670–1850, a period coinciding with intensive hunting. However, inbreeding among closely-related individuals may have occurred as recently as 10–20 years ago in the South cluster and on Öland. Consequently the low proportion of large ROH suggests that overharvesting did not impact inbreeding levels substantially or that the population recovered rapidly after the introduction of the new hunting regulation. Alternatively, gene flow and immigration from populations less impacted by hunting in eastern Europe or other parts of Scandinavia may have compensated for the strong bottleneck effect associated with overhunting^49^. While uncertainty around the recombination rate for moose can bias inference of the timing of inbreeding events, our estimate is consistent with the period of intense hunting of the 18^th^ to 19^th^ centuries and also reveals that contemporary moose populations may be potentially exposed to the negative effects of high inbreeding.

Additional genomes are also required to further examine the temporal dynamics of load in Swedish moose. Nevertheless, two competing hypotheses can explain the pattern observed here. In contrast with heterozygosity and inbreeding, modern genomes had a higher load than historical ones, suggesting that drift may have facilitated the increase in frequency of deleterious alleles during the near-extinction event. Alternatively, if an increase really occurred, drift alone may not have been sufficient to increase genetic load. We thus cannot exclude the possibility that new deleterious variation was introduced via gene flow from genetically distinct subpopulations during the recovery stage of the population.

When considering modern populations only, we observed an overall reduction in High and Moderate impact variants towards the South of the range. Higher inbreeding may have facilitated the exposure of deleterious variation in homozygous state (i.e., higher realised load) and their removal through purifying selection^32,33^. The moose population on Öland, which showed the highest inbreeding and a long-term small N_e_ over the past 2,000 years, did not have a significantly different total genetic load compared to the South cluster. However, we found a reduction in the frequency of High impact variants and an accumulation of Moderate impact ones. This result is consistent with theory and recent genomic studies (e.g.,^28,37,68^) showing that inbreeding facilitates the removal of highly deleterious variation through purifying selection, whereas mildly deleterious variation, with lower impact on fitness, tends to accumulate^16,32^. Temporal data would be required to formally examine the dynamics of purging on Öland.

Recent data supports a lower fitness on Öland, with calf survival rate being lower (~0.22) compared to other populations (~0.7)^69^. It has been hypothesised that poor body condition in females associated with suboptimal foraging habitat as well as a temporal mismatch between resource availability and parturition could explain this difference. However, our data suggest that higher exposure of deleterious alleles (i.e., higher inbreeding and realised load) and an excess in Moderate impact variants relative to the South cluster may also contribute to lower female conditions and lower calf survival. Furthermore, while Öland and the mainland may be connected by occasional gene flow^58^, which could lead to a genetic rescue effect, there is also a risk of the introduction of new deleterious variation from less inbred and more genetically diverse individuals from the mainland^70^. For instance, the migration of a single male into the highly inbred Isle Royal wolf (*Canis lupus*) population induced a fitness reduction associated with the introduction of new deleterious alleles^71^.

It is worth noting that various metrics can be used as proxies for genetic load and that these need to be interpreted carefully^16^. While the realised load affects individual fitness in the current generation, recessive deleterious mutations in heterozygote (i.e., masked load) state do not^16^. However, deleterious variants in heterozygous state are likely to be expressed in future generations if inbreeding increases, whereas outcrossing could mask homozygous variants into heterozygous state. The interpretation of these metrics is further complicated by the lack of information on selection (*s*) and dominance (*h*) coefficients, epistasis as well as variation in non-coding (e.g., regulating) regions^31^.

Genomic indicators raise concerns for the genetic health of Swedish moose. While N_e_ in the North and Transition clusters remain above acceptable levels, the South cluster exhibits N_eLD_ < 500, interpreted as a *Warning* due to the risk of losing adaptive and evolutionary potential. Additionally, inbreeding has increased and nucleotide diversity decreased over the past 150 years. However, additional historical and modern samples in this part of the range are required to confirm the observed temporal diversity patterns. Furthermore, population divergence has increased over time between the North and South clusters and between the North cluster and the Transition cluster, in both cases to such a degree that an *Alarm* is warranted for the F_ST_-indicator. This suggests that migration has decreased between genetic clusters, possibly due to higher hunting pressure and conflicts with humans (e.g., increased urbanisation and traffic-related deaths), presenting a risk of genetic erosion if populations become more isolated, especially in the South cluster.

Assessing the functional variation in wild populations is highly relevant to species monitoring^9,38^. Our genome scan analyses identified a number of outlier windows containing 206 genes under putative selection due to local adaptation, with 71 of those showing a significant association with latitude. The 206 candidate genes included functions associated with skin pigmentation, keratin synthesis, cardiovascular and nervous system. We also found 14 genes previously that are expressed in skin and horn of ungulates, including roe and sika deer antlers^56^. There is evidence for regional and habitat differences in moose antlers in Eurasia^72^ and North America^73^. For instance, in North America, moose from open tundra habitat have larger antlers than those from boreal forest (i.e., taiga)^73^. Yet, the relative genetic or diet type basis for such association is unclear^73^. Moreover, moose from mountain habitat in North America (*A. a. shirasi*) and Asia (*A. a. cameloides*) both have small body and antler size^74^. In Europe, moose from Sweden and Finland, distributed from open tundra habitat in the North to boreal forest in the South also show latitudinal variation in growth pattern and body size^53^ as well as an association between habitat and antler morphology, with the ‘palmated’ (i.e., webbed) type most prevalent in the North and the ‘cervina’ (i.e., deer-like) type most prevalent in the South^54^. Such association between habitat and morphology may thus indicate local adaptation and genes within the outlier windows identified here could thus be candidates for positive selection^75^.

Selective or trophy hunting may also induce allelic shifts involving genes associated with antler morphology as well as a reduction in antler size or even body size over time^76–78^. For instance, antler size in Alaskan moose has been reduced with increased harvest intensity^79^. Moreover, the Finnish moose population, which has been subjected to similar hunting pressures as the Swedish moose^80,81^, has experienced a rapid reduction in the ‘palmated’ type as a result of intensive hunting and/or fitness differences between antler types in managed forests between 1976 and 1999^54^. However, selective hunting alone may not be the only cause for horn size reduction and a combination of environmental factors, inbreeding depression and selective hunting may induce changes in male ornamentation (e.g.,^82^). When comparing 1980 and 2019/2020 genomes for the North cluster, our genome scan analysis identified a number of outlier windows containing genes associated with antler development^56^ as well as other functions (e.g., fertility, insulin and fat metabolism, cardiovascular system, body weight). Thus, over a short period of time (i.e., ca. five generations, assuming a generation time of seven years^49^), hunting pressure may not only have induced allelic changes in the vicinity of genes associated with male ornamentation but may have also impacted other types of functional variation with potential important fitness effects. Yet, strong drift during the bottlenecks can also induce changes in the frequency of certain alleles and thus result in genome-wide differentiation which could be interpreted as selection^83^. For instance, it has been suggested that the high number of outlier regions among populations of three-spine stickleback (*Gasterosteus aculeatus*) from different environments may have arisen due to repeated bottlenecks and strong drift from small lake populations^83,84^.

In conclusion, our study suggests that while the near-extinction of moose in Sweden did not severely impact its genome-wide diversity, at least in terms of inbreeding, our modern data raises concerns that reduction in gene flow, sudden declines and hunting practices may negatively impact the functional variation of moose populations. At present, the indicators proposed by the CBD do not include estimates of adaptive or deleterious variation^26,27^. We thus advocate for the inclusion of metrics estimating both deleterious and selectively advantageous variation in genetic monitoring programmes. Doing so will also allow the incorporation of genetic metrics with more relevance to species survival within the IUCN Red List^15,85^ and Green Status of Species^86^.’ Our study thus illustrates the need for continued genomic monitoring using genomic indicators and to incorporate estimates of deleterious and adaptive variation for the effective management of wild species.

## Methods

### Ethical statement

The moose samples used for the assembly generation were obtained from frozen tissue banks collection from 1980 maintained by L.L. and N.R. at Stockholm University and from 2019/2020 maintained by G.E. and G.S. at Umeå University. No Ethics approval was required.

### Sampling, DNA extraction and library preparation

We extracted DNA from a total of 87 genomes (57 from 1980, 23 from 2019/2020, and seven historical genomes from 1839–1905; Supplementary Data 2) representing the whole geographical distribution of moose in Sweden (Fig. 1a). Modern DNA was extracted from ~20 mg of muscle tissue using a DNeasy Blood & Tissue Kit (Qiagen, Hilden, Germany) and historical DNA from antlers or jawbone following Yang et al.^87^.

Genomic library preparation from modern DNA extracts was performed using a PCR-free protocol at the Science for Life Laboratories (SciLifeLab), Stockholm. Genomic libraries for historical DNA were prepared as described in Dussex et al.^88^, following Meyer & Kircher^89^ and including USER treatment to remove deaminated bases^90^ (and thus sequencing error) and five independent PCRs were run to increase complexity. Libraries were sequenced on a NovaSeq S4 flowcell using a 2x150bp and 2x100bp setup for modern and historical libraries, respectively.

### De-novo assembly

We improved the assembly from Dussex et al.^49^ (GenBank accession: GCA_015832495.1) by using Hi-C scaffolding. In brief, 36 mg of fresh frozen muscle tissue from the same female individual from central Sweden (Province of Gävleborg) was ground to a fine powder and used as input for Omni-C™ Proximity Ligation Assay, Mammalian Samples Protocol version 1.2 from Cantata Bio according to the specifications from the manufacturer. The resulting library was pooled and sequenced to approximately 25% lane capacity on an Illumina NovaSeq 6000 instrument using S4 Reagent Kit v1.5 (300 cycles). The scaffolding was performed following Dudchenko et al.^91^, which includes Juicer (v1.6) for read-mapping and filtering, 3D-DNA (v180922) for draft scaffolding and Juicebox (v1.11.08) for manual curation of Hi-C scaffolds. For assembly evaluation we used QUAST (v5.0.2)^92^ and BUSCO (v. 5.3.1)^93^ with the “mammalia_odb10” lineage dataset.

We identified repeats using repeatmodeller v1.0.11 and repeatmasker v4.0.7 [Smit, A.F.A. and Hubley, R. (2008–2015) RepeatModeler Open-1.0, http://www.repeatmasker.org/] and CpG sites using the GenErode bioinformatics pipeline^94^. Finally, we used Minimap2^95^ to perform a synteny analysis against red deer (*Cervus elaphus;* Genbank ID: GCA_002197005.1) and identify sex-linked scaffolds. Since we also mapped the data to *O. hemionus* for the SNPeff and genome scan analyses, we used the same approach to identify the X chromosomes (HiC_scaffold_35) in *O. hemionus*.

### Modern and historical data mapping

Mapping and variant calling of modern and historical genomes was done using the GenErode bioinformatics pipeline^94^. Briefly, adapter trimming was done with fastp v0.22.0^96^ for modern data while we used Seqprep v1.1 (https://github.com/jstjohn/SeqPrep) to trim and merge forward and reverse reads for historical data. Reads for modern and historical data were then mapped using BWA v0.7.17 mem and aln algorithms, respectively, while sorting and removing PCR duplicates were done using SAMtools v1.12. Finally, reads were realigned around indels using GATK IndelRealigner v3.4.0^97^.

Variant calling was done with the mpileup command of bcftools v1.8^98,99^. We used a minimum depth of coverage (DP4) of ~1/3 (i.e., 5X) of the average depth of coverage, and filtered SNPs by base quality QV ≥ 30 and those within 5 bp of indels. Sites with SNPs in heterozygous state were filtered out if the allele frequency fell outside an allelic balance (i.e., number of reads displaying the reference allele/depth) of <0.2 and >0.8 in order to avoid biases caused by contamination, mapping or sequencing error. After merging all individual vcf files, we masked CpG sites using BEDtools v2.27.1^100^ to limit possible biases from DNA damage in historical genomes. We also masked repeat sites with BEDtools, and excluded scaffolds linked to X and Y chromosomes. Finally, we examined DNA damage patterns for the seven historical genomes using MapDamage^101^. The USER treatment removed the majority of typical post-mortem damage (Supplementary Fig. 5).

We obtained a total of 4,265,381 high-quality SNPs across our 87 genomes. For all downstream analyses involving population comparisons, we used PLINK v2^102^ to retain only SNPs called in all 87 individuals, making for a total of 617,661 SNPs.

For analyses that required the calling of variants relative to an ancestral allele (e.g., SnpEff, PBS), we mapped the data as described above against mule deer *Odocoileus hemionus*; Odocoileus_hemionus_HiC, https://www.dnazoo.org/assemblies/Odocoileus_hemionus). After filtering for missing data and excluding the sex chromosome, we obtained a total of 5,305,454 SNPs.

### Population structure

We performed an ADMIXTURE v1.3.0^103^ analysis to estimate individual-based ancestry and identify genetic clusters (K = 1–5). This approach assumes that individuals are unrelated and uses a cross-validation procedure to determine the best number of possible genetic groups present in the dataset.

We estimated pairwise F_ST_^104^ between the North and South clusters as well as between the North and Transition clusters for each time period (i.e., historical, 1980, 2019/2020) with VCFTools v0.1.16^105^ using non-overlapping windows of size 50 kb (see also *Genomic indicators* section).

### Past demography

To estimate the recent past demography (i.e., <10,000 years BP), we used the SMC++ v.1.15.2^106^. Like PSMC^107^, this approach relies on Sequential Markov Coalescent (SMC) simulations from unphased genome data from multiple genomes instead of one single diploid genome, thereby increasing the number of recent coalescent events to estimate the effective population size (N_e_). Using the same substitution rate and generation time as for the PSMC from Dussex et al.^49^ (i.e., 7 years; 7 × 10e^−9^), past demography was estimated using the ‘cross validation’ approach with --em-iterations 5000, and --thinning 1300 and --regularisation-penalty 6.

We also examined the past demography of moose over the past 100–200 generations using GONE^60^ which estimates changes in N_eLD_ calculated as the geometric mean over 40 independent estimates from the observed spectrum of linkage disequilibrium (LD; https://github.com/esrud/GONE). We only retained the 32 largest autosomal chromosomes and used the following parameters: PHASE = 2; cMMb=1; DIST = 1; NGEN = 2000; NBIN = 400; MAF = 0.0; ZERO = 1; maxNCHROM=85; maxNSP = 50000; hc = 0.05; REPS = 40; threads = −99. For both of these analyses, historical genomes were excluded. We performed this analysis separately for the three genetic clusters identified by Wennerström et al.^48^ and the island population of Öland to avoid any underestimation caused by population substructure. We also used a generation time of seven years^49^.

### Heterozygosity, genome-wide diversity and inbreeding

We estimated heterozygosity using mlRho v2.7^108^ which estimates the individual mutation rate (θ). Under the infinite sites model, θ approximates the genome-wide heterozygosity measured as the number of heterozygous sites per 1,000 bp. We downsampled each genome to the average coverage of the genome with lowest coverage (i.e., 7X), filtered out bases with quality (-Q) < 30, mapped sequencing reads with mapping quality (-q) <30 and positions with root-mean-square mapping quality (MQ) < 30 from the historical and modern bam files. We then filtered out sites with depth <1/3 (i.e., 5X) of the average depth of coverage. We also estimated nucleotide diversity (*π*) per population and time point using VCFTools v0.1.16 and with 50 kb non-overlapping windows.

Inbreeding coefficients (F_ROH_) were estimated based on Runs of Homozygosity (ROH) that were identified using the sliding-window approach implemented in PLINK v1.9. We used a set of strict parameters to avoid overestimation and overrepresentation of long ROH: a sliding window size of 100 (homozyg-window-snp 100); no more than 1 site per window to assume a window as homozygous (homozyg-window-het 1); at least 5% of all windows including a given SNP to define the SNP as being in a homozygous segment (homozyg-window-threshold 0.05); a homozygous segment was defined as a ROH if the segment included ≥25 SNPs (homozyg-snp 25) and covered ≥100 kb (homozyg-kb 100); the minimum SNP density was one SNP per 50 kb (homozyg-density 50); and the maximum distance between two neighbouring SNPs was ≤1,000 kb (homozyg-gap 1,000). Finally, we set the value at 750 heterozygous sites within ROH (homozyg-het 750) in order to prevent sequencing errors to cut ROH. The inbreeding coefficient F_ROH_ was then estimated as the overall proportion of the genome (autosomes only) comprising ROH. We statistically tested for differences in heterozygosity and F_ROH_ among modern populations using ANOVAs and used Tukey’s HSD tests to test for pairwise differences among time periods for each population and among modern populations in R^109^.

Using the length of ROH, we also estimated the timing of inbreeding events using:

1 $$g=100/(2{rL})$$

where *g* corresponds to the number of generations, *L* to the length of ROH in Mb, and *r* to the recombination rate^67^. For the latter we used an estimate of 1.04 cM/Mb estimated in red deer^110^. Based on these parameters and using a generation time of seven years^49^, ROH ≥ 0.5, ≥2, ≥5, ≥10 and ≥30 correspond to inbreeding events occurring ca. 96 (670 years), 24 (168 years), 9.6 (67 years), 4.8 (33 years) and 1.6 generations (11 years) before present (BP), respectively.

### Genetic load

We used SnpEff v4.3^111^ to annotate synonymous and non-synonymous nucleotide substitutions in coding regions. All genomes were mapped against mule deer (*Odocoileus hemionus*; Odocoileus_hemionus_HiC) to reduce reference mapping and annotation bias. After removing gene models with in-frame STOP codons from the annotation (Odocoileus_hemionus_HiC.fasta_v2.functional.gff3), missing START and terminal STOP codons (-J option) and genes labelled as pseudogenes (--no-pseudo option) with Cufflinks v2.2.1^112^, we obtained a total of 22,732 genes.

Next, we generated a database for *O. hemionus* using the protein sequences extracted from the annotation. We first identified putative deleterious variants by allocating variants to three different impact categories as defined in the SnpEff manual: a) Low/Synonymous: mostly harmless or unlikely to change protein behaviour; b) Moderate: non-disruptive variants that might change protein effectiveness; c) High: variants assumed to have high (disruptive) impact on protein, probably causing protein truncation, loss of function or triggering nonsense-mediated decay and including stop gained codons, splice donor variant and splice acceptor, start codon lost^111^. We also excluded intergenic (-no-intergenic) and intron (-no-intron) variants. We estimated individual genetic load in two ways. First, after removing variants fixed in all individuals, we estimated the total individual load by summing the number of variants of each category *i*. We also corrected for potential mapping biases dividing the number of each category by the total number of synonymous SNPs, following Xue et al.^113^. Secondly, we estimated the individual realised load (i.e., total number of homozygous variants of category *i* divided by twice the total number of segregating sites for category *i*^114^). By taking into account the mode of dominance, this estimate allows us to estimate the proportion of potential load that is realised or expressed in each individual. We statistically tested for differences in genetic load among modern populations using ANOVAs and used Tukey’s HSD tests to test for pairwise differences among time periods for each population and among modern populations in R^109^.

To take into account the frequency of variants in each population, we also calculated the R_xy_ ratio of Moderate and High impact variants for modern population pairs and for temporal comparisons following von Seth et al.^115^. An R_xy_ equal to 1 corresponds to no change in frequency between two populations, whereas R_xy_ < 1 or >1 corresponds to a decrease/deficiency or an increase/excess in frequency in population x relative to population y, respectively. We used a jack-knife procedure in R^109^ to estimate the variance in the ratio.

### Genomic indicators

Three indicators to monitor trends in genetic diversity have recently been introduced in Swedish monitoring work following science-management elaborations^24^. The indicators are based on standard population genetic metrics for within and between population variation and agree with Essential Biodiversity Variables (EBVs) for genetic diversity^26^ and are also relevant for the indicator assessments required by the new monitoring framework of the Convention on Biological Diversity (CBD)^27^. Two indicators assess diversity within populations (ΔH and N_e_)^20,24^. The third indicator (ΔF_ST_) reflects temporal changes in genetic diversity among populations as well as the number of populations maintained over time.

The ΔH indicator uses several measures of within population diversity and was estimated for the three clusters, with each cluster stratified into separate time points based on available samples. For the ΔH indicator, we used ΔHET, Δπ, and ΔF_ROH_ based on our estimates of heterozygosity, nucleotide diversity, and inbreeding (see above). For the North cluster, three temporal stratifications were possible; 1800s, 1980, and 2019/2020. Two stratifications were made for each of the remaining two clusters; 1980 and 2019/2020 for the Transition cluster and for the South cluster 1800s and 1980. For the N_e_ indicator, we used estimates from the GONE analysis from the most recent generation (i.e., 1 generation BP; Supplementary Data 3). The ΔF_ST_ indicator requires F_ST_ between at least two populations at two points in time to be calculated and was thus only possible for two temporal comparisons: F_ST_ between North and South clusters in the 1800s and in 1980, and between the North cluster and the Transition zone in 1980 and 2019/2020.

Next, for each indicator, threshold values reflecting specific rates of change over time were used to assess trends in genetic diversity of populations (see Fig. 3 in Andersson et al.^24^). Three indicator signals (i.e., *Alarm* / *Warning* / *Acceptable*) were given. For the ΔH indicator, and in cases of significant temporal change, the difference in diversity between two time points over the whole sampling period (between 40 and 170 years for our different comparisons) was translated into an annual change. The threshold values for annual reduction (≤0.05%; *Acceptable*, 0.06–0.3%; *Warning*, >0.3%; *Alarm*) were applied to the three measures of genomic diversity (i.e., heterozygosity, π, and F_ROH_). For the N_e_ indicator, N_eLD_ ≥ 500 is classified as *Acceptable*^116^, 50< N_eLD_ < 500 *Warning*, and N_eLD_ < 50 *Alarm* based on^116,117^.

The ΔF_ST_ indicator was classified as *Acceptable* if no statistical change was detected. In cases of a significant temporal change, F_ST_ was converted to gene flow between clusters (i.e., expected number of migrants^24^). A ΔF_ST_ reflecting an increase or decrease in migration was classified as follows: reduced migration by 25% and 50% was considered *Warning* and *Alarm*, respectively and increased migration of 50% and 100% classified as *Warning* and *Alarm*, respectively. This indicator was also classified as *Alarm* if any population went extinct over the monitoring period.

Only statistically significant changes in genetic metrics were considered^24^ and statistical testing of indicators was performed in R^109^ using *t*-tests and Wilcoxon signed-rank tests. Only results from the t-test are reported in cases where these two tests are concordant with each other.

### Genomic differentiation and genome scans of selection

To identify candidate regions under putative positive selection and potentially due to local adaptation, we estimated pairwise F_ST_ between the North (*n* = 30) and South (*n* = 30) clusters in ANGSD^118^. We first calculated the sample allele frequency (saf) for the data mapped to O. *hemionus* with the following arguments: -doSaf 1; -gl 1; -minMapQ 30; -minQ 30. We used *O. hemionus* as reference for the ancestral state to estimate the unfolded Site Frequency Spectrum (SFS). Next, we calculated the SFS using the *realSFS* command to calculate 2d sfs for the North vs South populations comparison using the following options: -P 10; -r. We then used the *realSFS fst index* command to calculate F_st_ binary files. We used the *realSFS* command to extract F_st_ values and a sliding with the following options: -win 50,000; -step 10000. We performed a Z-transformation of F_ST_ values and retained F_ST_-outlier windows with a Z-score >5 (i.e., values outside 5 standard deviations from the mean^119^). Z(F_ST_) was calculated as Z(F_ST_) = (F_ST_ – μ F_ST_)/σ F_ST_, where F_ST_ is the F_ST_ value in a window, μ F_ST_ the average F_ST_ over all windows, and σ F_ST_ the standard deviation of F_ST_ values over all windows. Finally, we extracted those Z(F_ST_) outlier windows as a bed file and cross-referenced them with the annotation for *O. hemionus* (Odocoileus_hemionus_HiC.fasta_v2.functional.gff3) using BEDtools intersect^100^.

In order to test for association between the identified outlier windows and climate, we used a Latent Factor Mixed Model (LFMM) implemented in the LEA R package^120^. We used BEDtools intersect to extract the variants that were overlapping with Z(F_ST_) outlier windows from our 80 modern genomes vcf file (data mapped to *O. hemionus*). Latitude was used as a proxy for temperature and as the explanatory variable in the model. We ran the model with K = 3 and for 20 repetitions. Significance was applied using an alpha value of 0.001 and a Bonferroni correction for multiple testing. We then extracted candidate SNPs and cross-referenced them with the annotation for *O. hemionus* using BEDtools intersect.

Finally, we estimated pairwise F_ST_ between the 1980 (*n* = 17) and 2019/2020 (*n* = 17) periods for the North cluster to identify recent temporal genomic differentiation as described above. This analysis was only performed for this cluster since it was the only one for which sample size was large enough for a temporal comparison and since substructure over the moose range could bias our results.

### Gene ontology

For all genes identified with the genome scans analyses, we used the Mouse Genome Informatics database (www.informatics.jax.org) to manually retrieve gene ontologies and mammalian phenotype information for each candidate gene. We also compared our list of candidate genes with that of Wang et al.^56^.

### Statistics and reproducibility

The research sample included 80 modern and 7 historical moose samples collected across the whole range of the species in Sweden. All statistical tests were conducted using publicly available programs and packages as described in the methodological sections above. Reproducibility can be accomplished by following the laboratory and analytical protocols described above and by following. The author’s GitHub (https://github.com/ndussex/Moose_genomics) contains details on the code and analyses used in this study.

### Reporting summary

Further information on research design is available in the Nature Portfolio Reporting Summary linked to this article.

### Supplementary information

Supplementary Information

Description of Additional Supplementary Files

Supplementary Data

Reporting Summary

## Data Availability

Assembly: Genbank (BioProject: PRJNA668262; Accession number: GCA_015832495.2; NRM_Aalces_2_0.fsa; JADEYB000000000). Resequencing data: PRJEB60841. See Supplementary Data 2 for estimates of Heterozygosity, inbreeding and genetic load.

## Code availability

The full code used for the population genomic analyses is available on: https://github.com/ndussex/Moose_genomics.
