# Supplementary material for: Range-wide and temporal genomic analyses reveal the consequences of near-extinction in Swedish moose
Source: Commun Biol. 2023 Oct 17;6:1035. doi: 10.1038/s42003-023-05385-x (PMC10582009; doi:10.1038/s42003-023-05385-x)
Supplement: Supplementary file 1 — Supplementary Information [file 42003_2023_5385_MOESM1_ESM.pdf]

Supplementary material

Range-wide and temporal genomic analyses reveal the consequences of near-extinction in Swedish moose

Nicolas Dussex[1,2,3,4], Sara Kurland[2], Remi-André Olsen[5], Göran Spong[6], Göran Ericsson[6], Robert Ekblom[7], Nils Ryman[2], Love Dalén[1,2], Linda Laikre[2]

1. Centre for Palaeogenetics, Svante Arrhenius väg 20C, SE-106 91 Stockholm, Sweden
2. Department of Zoology, Division of Population Genetics, Stockholm University, SE-106 91 Stockholm, Sweden
3. Department of Bioinformatics and Genetics, Swedish Museum of Natural History, Stockholm, Sweden
4. Norwegian University of Science and Technology, University Museum, Trondheim 7491, Norway
5. Science for Life Laboratory, Department of Biochemistry and Biophysics, Stockholm University, SE-17121 Solna, Sweden
6. Department ofWildlife, Fish, and Environmental Studies, Swedish University ofAgricultural Sciences, Umeå, Sweden
7. Wildlife Analysis Unit, Swedish Environmental Protection Agency, SE-10648 Stockholm, Sweden

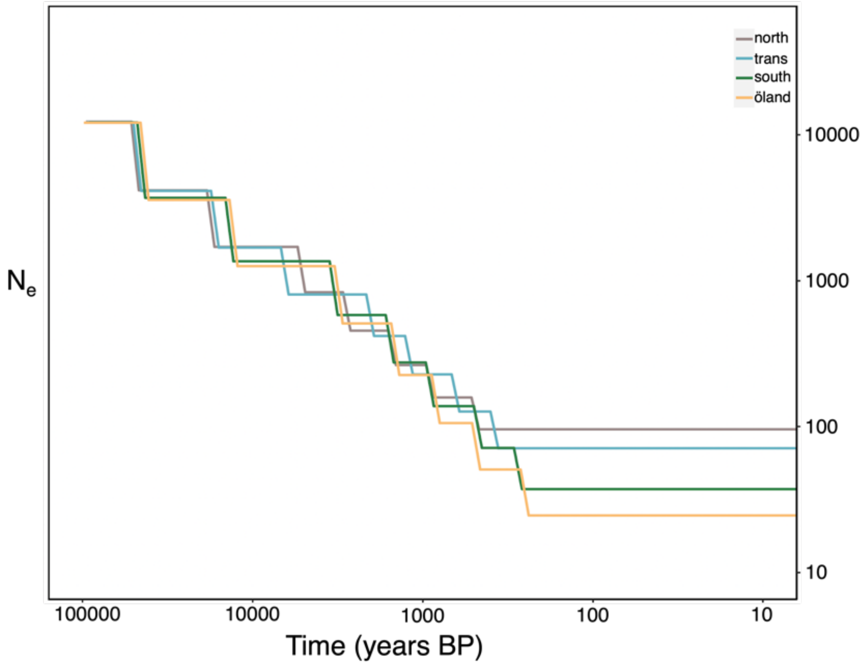

Supplementary Figure 1. Demographic reconstruction of effective population size (N_e) SMC++ using the cross-validation procedure.

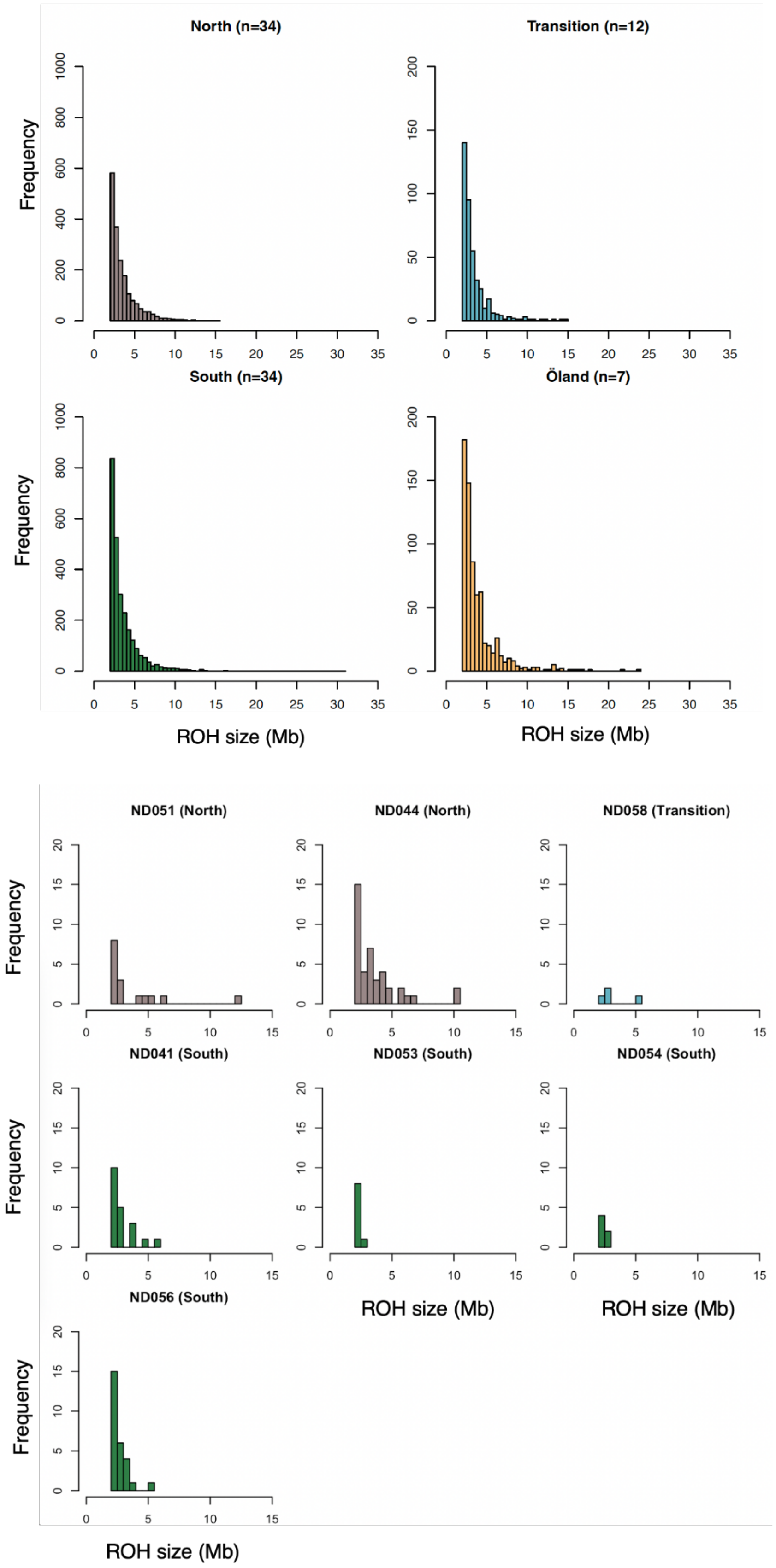

Supplementary Figure 2. ROH length distribution per population and for the seven historical genomes. Only ROH ≥2Mb are shown.

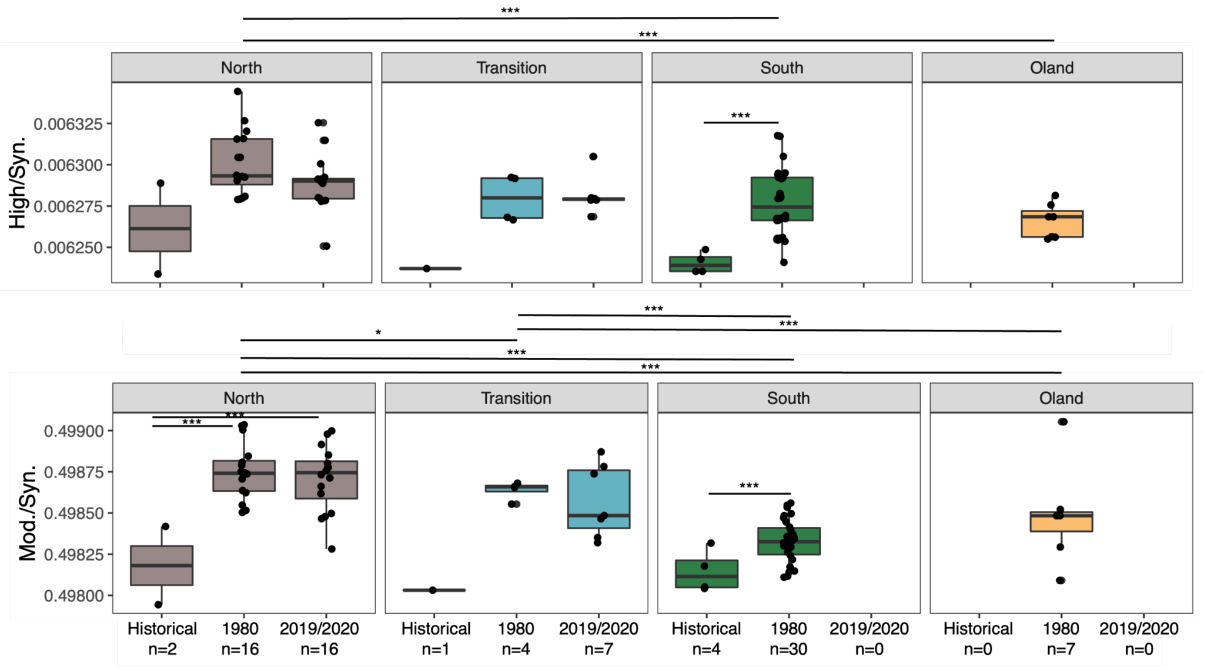

Supplementary Figure 3. High and Moderate variants to Synonymous ratio.

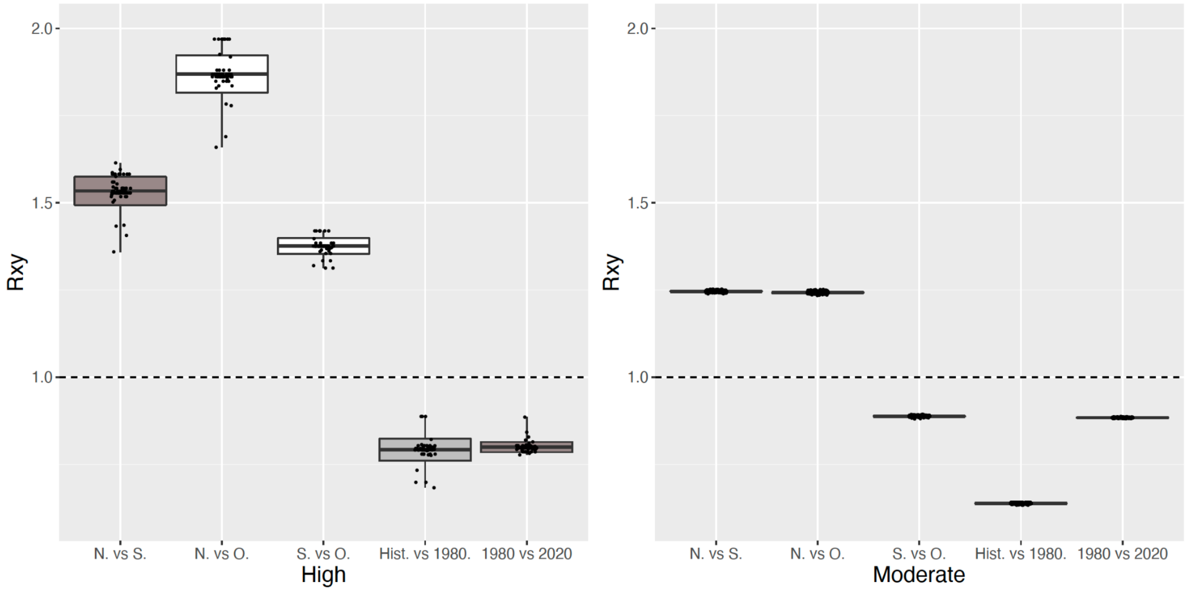

Supplementary Figure 4. R_{xy} of derived alleles for Moderate and High impact variants for Swedish moose. R_{xy} >1 or <1 indicates a relative frequency increase or decrease in population x vs y, respectively, for a given variant category (N. = North; S. = South; O. = Öland, Hist- = Historical; 80s = 1980; 19/20 = 2019/2020). Horizontal lines within boxplots represent the mean, bounds of boxes represent the standard deviation and vertical bars represent minima and maxima.

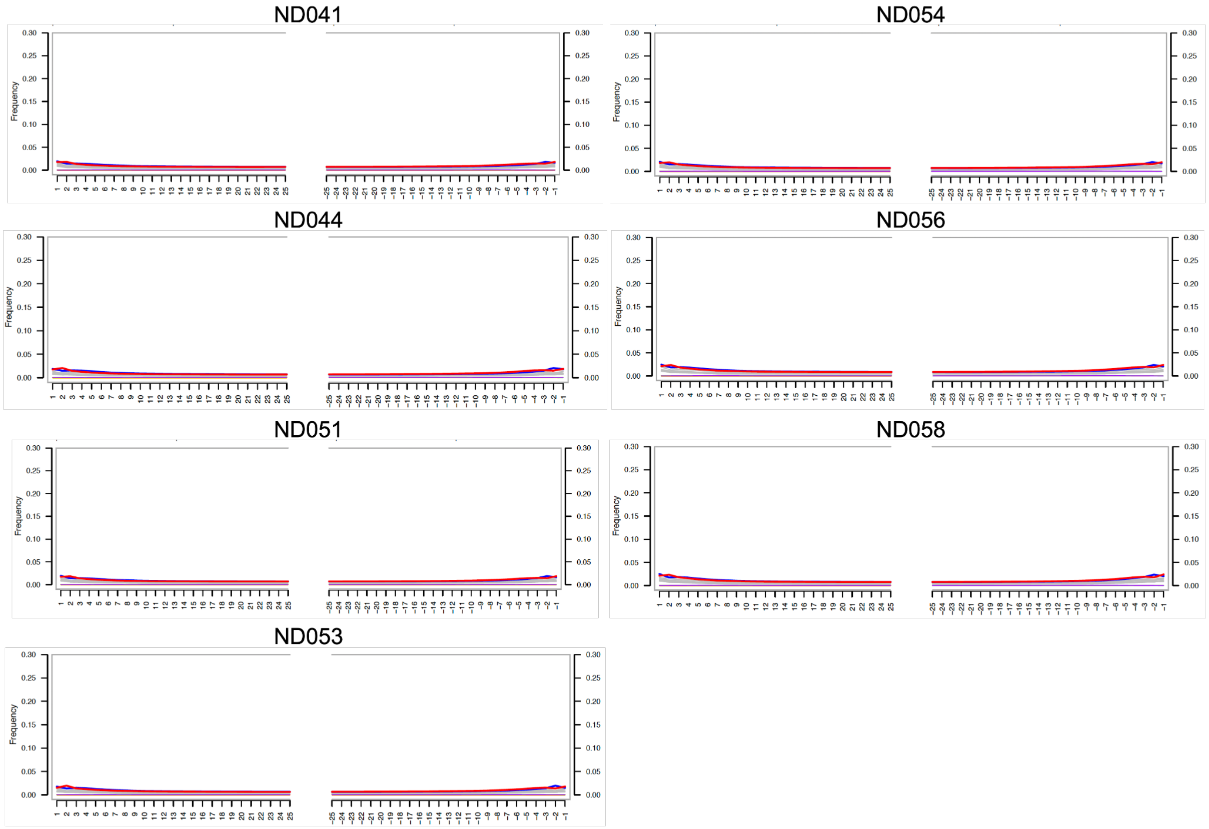

Supplementary Figure 5. Fraction of C-to-T substitutions by distance from the read-end for the seven historical moose genomes. Minimal to no post-mortem damage is observed due to USER treatment.
