# Supplementary material for: Range-wide and temporal genomic analyses reveal the consequences of near-extinction in Swedish moose
Source: Commun Biol. 2023 Oct 17;6:1035. doi: 10.1038/s42003-023-05385-x (PMC10582009; doi:10.1038/s42003-023-05385-x)
Supplement: Supplementary file 2 — Description of Additional Supplementary Files [file 42003_2023_5385_MOESM2_ESM.pdf]

Description of Additional Supplementary Files

File name: Supplementary Data 1
Description: Scaffold list

File name: Supplementary Data 2
Description: Sample metadata and genetic estimates

File name: Supplementary Data 3
Description: Temporal genome-wide diversity within genetic clusters

File name: Supplementary Data 4
Description: Temporal effective population size estimates

File name: Supplementary Data 5
Description: High and Moderate impact frequencies of variants

File name: Supplementary Data 6
Description: Gene functions associated with the genetic load analysis

File name: Supplementary Data 7
Description: Genetic indicator classifications within populations

File name: Supplementary Data 8
Description: Genetic indicator classifications between populations

File name: Supplementary Data 9
Description: Candiate genes based on the Z(FST) outlier analysis

File name: Supplementary Data 10
Description: Gene attributes for the candidate outlier genes
